# 3D Printed porous polyamide macrocapsule combined with alginate microcapsules for safer cell-based therapies

**DOI:** 10.1038/s41598-018-26869-5

**Published:** 2018-05-31

**Authors:** Laura Saenz del Burgo, Jesús Ciriza, Albert Espona-Noguera, Xavi Illa, Enric Cabruja, Gorka Orive, Rosa María Hernández, Rosa Villa, Jose Luis Pedraz, Mar Alvarez

**Affiliations:** 1 NanoBioCel Group, Laboratory of Pharmaceutics, School of Pharmacy, University of the Basque Country (UPV/EHU), Paseo de la Universidad 7, 01006 Vitoria-Gasteiz, Spain; 2 Instituto de Microelectronica de Barcelona (IMB-CNM, CSIC), Campus UAB, 08193 Bellaterra, Barcelona, Spain; 3 Biomedical Research Networking Center in Bioengineering, Biomaterials and Nanomedicine (CIBER-BBN), Madrid, Spain

## Abstract

Cell microencapsulation is an attractive strategy for cell-based therapies that allows the implantation of genetically engineered cells and the continuous delivery of *de novo* produced therapeutic products. However, the establishment of a way to retrieve the implanted encapsulated cells in case the treatment needs to be halted or when cells need to be renewed is still a big challenge. The combination of micro and macroencapsulation approaches could provide the requirements to achieve a proper immunoisolation, while maintaining the cells localized into the body. We present the development and characterization of a porous implantable macrocapsule device for the loading of microencapsulated cells. The device was fabricated in polyamide by selective laser sintering (SLS), with controlled porosity defined by the design and the sintering conditions. Two types of microencapsulated cells were tested in order to evaluate the suitability of this device; erythropoietin (EPO) producing C_2_C_12_ myoblasts and Vascular Endothelial Growth Factor (VEGF) producing BHK fibroblasts. Results showed that, even if the metabolic activity of these cells decreased over time, the levels of therapeutic protein that were produced and, importantly, released to the media were stable.

## Introduction

Bioactive cell encapsulation has emerged as a promising tool for the treatment of patients with various chronic disorders including diabetes mellitus, central nervous system diseases, and cardiovascular diseases^1–4^. In fact, cell encapsulation is one of the current leading methodologies aimed at the immobilization of allogeneic or xenogeneic cells in a semipermeable but immunoprotective membrane to deliver biological products to patients. Thus, the implantation of primary cells, stem cells or *ex vivo* genetically modified microencapsulated cells provides a possible alternative for the continuous delivery of recombinant proteins. At the same time, this approach provides a physical barrier to mask the implant from the host´s immune surveillance following *in vivo* implantation without the need for systemic immunosuppression, as it prevents direct cell-to-cell contact and thus, avoids the activation of cytotoxic CD8^+^ T cells^5–7^. One of the main drawbacks of microcapsules implantation is their dissemination through the surroundings of the implanted area of the body, complicating the microcapsules removal if required, either when cells need to be renewed, the therapy provokes severe side effects in the patient, or once the therapy reaches its goal. Therefore, it is critical to devise systems to maintain long-term cell survival and consistent production of the therapeutic factor, but, at the same time, allow the retrieval of the implanted cells.

Macroencapsulation devices are a promising approach to solve this limitation as they can maintain the encapsulated cells in a known location and, importantly, they can improve even more the immunoisolation of the cells^8,9^. The materials used for the development of macro-devices are mechanically more stable than the ones used for microencapsulation, and therefore, they are more secure. However, these devices for clinical applications have to meet essential requirements. In fact, the overall device geometry would determine the cell content and, therefore, the amount of therapeutic molecule that would be secreted^10,11^. Also, encapsulated cells need to be alive in the long-term and this, importantly, relies on the supply of oxygen and nutrients^12^. Because oxygen diffusion is slower than oxygen consumption, this is the limiting factor in cell survival. The progress in macroencapsulation has been limited due to, on the one hand, the inefficient mass transport of oxygen and nutrients under extravascular setting, and, on the other hand, problematic blood coagulation and thrombosis under intravascular environments because the device is implanted into the vessels of the host by vascular anastomoses^13–15^. In addition, healing in the presence of synthetic medical devices is known to dramatically differ from normal wound healing, particularly due to the occurrence of chronic inflammation. Thus, in order to design a system that can be translated into the clinic, it is essential to use materials that comply with the specifications imposed by regulatory agencies for medical devices.

Currently, a small number of encapsulation systems have been applied clinically, and most of them have been aimed to recover endocrine pancreatic function (Encaptra, βAir, Sernova cell pouch). However, this technology has opened a broad range of potential applications, reaching the clinical trial on the eye disease treatment (Neurotech) or the central nervous system (NTCELL® for Parkinson’s disease). Both, Neurotech and NTCELL, are based on an immunoisolating hollow-fiber membrane (NT device) with an internal scaffold and hold a phase II clinical study. The Encaptra device for subcutaneous implantation, commercialized by ViaCite, is as well based on a single immunoprotective membrane with a small pore size, and is currently under phase I/II clinical trials in combination with stem-cell technology. The main drawback of this approach based on small pore size membranes is the poor oxygen and nutrients exchange that may affect the cell viability. At the same time, strategies for subcutaneous transplantation, which provides ready access to the graft, often fail because of the foreign-body inflammatory reaction and the formation of a fibrotic tissue around the graft^16^. The engineering of biomaterials could further improve neovascularization while minimizing fibrotic capsule formation^17^. The use of growth factors has as well demonstrated to increase the cell viability by achieving a faster revascularization^18,19^. Other approaches relay on the pre-vascularization of larger pore size (∼5 µm) polymeric implanted devices^20,21^, or cavities^22,23^, before cells allocation, combined with the use of systemic immunosuppression to prevent the host immune system from attacking the transplanted cells. With this approach, Theracyte device and the Sernova Cell Pouch, have reached clinical trials to restore pancreatic function^8,24^. More recently, 3D printing techniques have become a promising, cheap, and simple tool for scaffold and devices fabrication by using biocompatible materials with on-demand design and porosity^25,26^.

**Figure 1:**
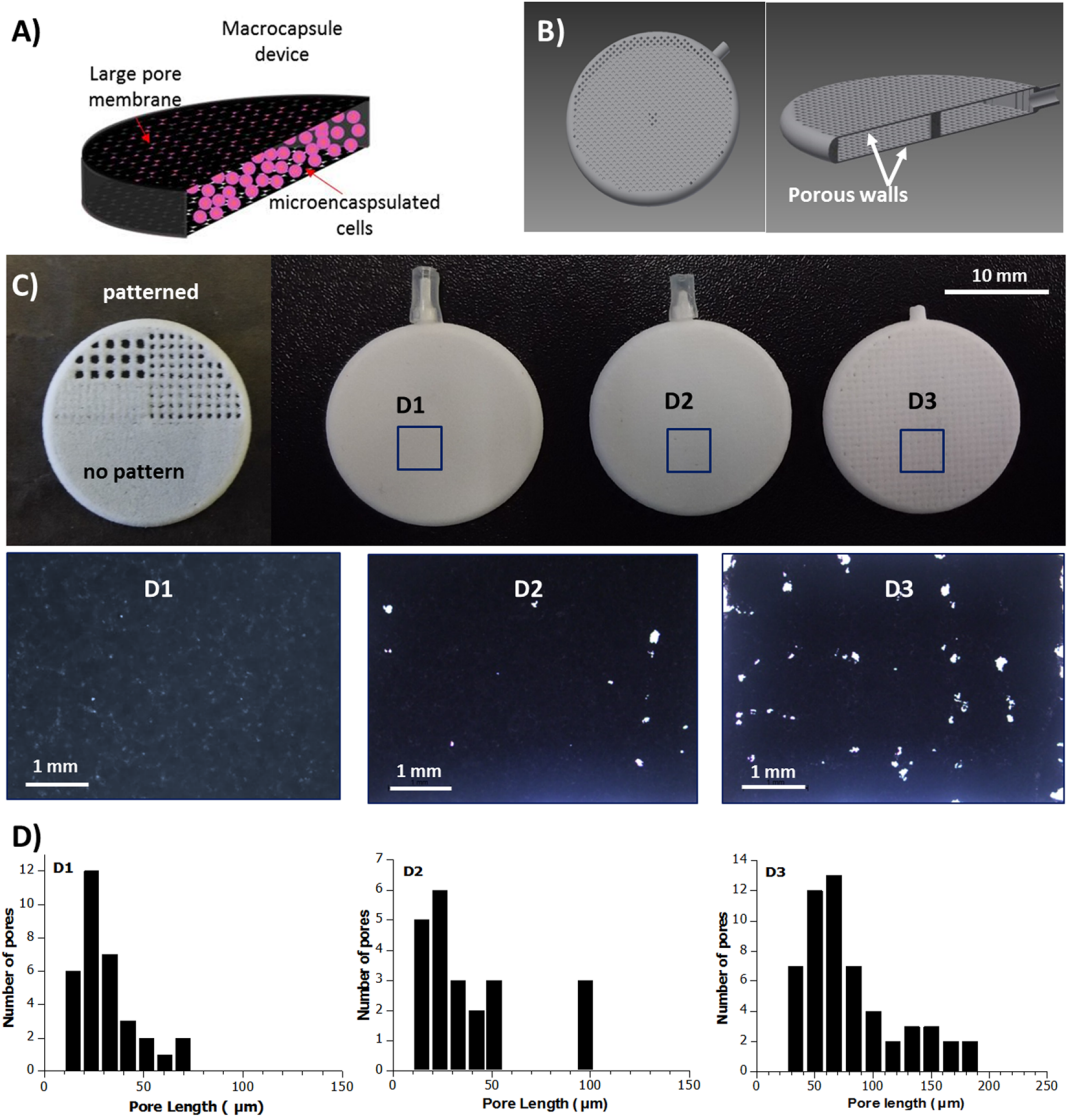
(**A**) Sckeme of the double encapsulation approach (not at scale). (**B**) CAD design image of the device showing a hole and cross-sectioned device. (**B**) SLS fabricated test pattern (half-device) and 3D macrocapsule devices fabricated with different pore sizewith their corresponding optical images of the macrocapsule wall. (**D**) Pore length histograms.

In contrast with the retrievable macroscale encapsulation devices mentioned before, in this work we propose the use of a double encapsulation barrier by combining the cell microencapsulation technology with the use of an external macro-device with an optimum larger pore size to enclose the microcapsules. The alginate-poly-L-lysine-alginate (APA) microcapsules would assure a correct immunoisolation when used *in vivo*. The molecular weight cut-off of this semi-permeable membrane lies in approximately 60–70 KDa, and, therefore, the entrance of IgG and IgM antibodies of the humoral immunity as well as the cells involved in the cellular immunity response are blocked. However, lower molecules necessary for cell survival such as glucose (180 Da) and carbon dioxide (44 Da) and secretary proteins from cells such as growth factors (6–50 KDa) can easily pass^27,28^. On the other hand, the external macro-device would maintain the total volume of the loaded microcapsules in the implantation site (Fig. 1A). The large size of the alginate microencapsulated cells (∼400 µm) respect to the non-encapsulated cells allows the fabrication of an external macro-device with a larger pore size, promoting the oxygen and nutrients exchange and probably the vascularization process. Avoiding the requirement of small pore size on the macrocapsule device opens the window to the use of 3D printing techniques with the ability to rapidly prototype different device designs and covering a new range of biocompatible materials. We fabricated polymeric implantable 3D devices, with controlled pore size, by using selective laser sintering (SLS), to allocate previously manufactured alginate-poly-L-lysine microcapsules containing either erythropoietin producing C_2_C_12_ myoblasts (C_2_C_12_-EPO) or VEGF producing BHK fibroblasts (BHK-VEGF). Polyamide was chosen for the macro-device fabrication because it’s demonstrated biocompatibility and excellent mechanical properties (high mechanical strength and high flexibility). We evaluated the oxygen and nutrients exchange through the device membranes and the biocompatibility of the devices according to the international standards. Then, we deeply analyzed by different means the biological behavior of encapsulated cells and the production and release of the therapeutic proteins produced by the cells.

## Results and Discussion

### Macrocapsule design, fabrication and characterization

One of the bottlenecks in macro-encapsulation strategies is the large diffusion distance from neighboring blood vessels to the encapsulated cells (decreasing the oxygen and nutrients supply) and consequent reduction of long-term cell survival and functioning. To surpass this problem, we chose a hollow thin disc design, looking to increase the device surface area and, therefore, increase the number of microcapsules in contact with the macrocapsule walls, avoiding the formation of several layers of microcapsules deep inside the macrocapsule where the oxygen and nutrients diffusion would be worse. Larger surface area would means larger vascularization area and, then, larger transport of oxygen and nutrient inside the device. The thickness of the macrocapsule walls (membrane) corresponds to a single sintered layer, of about 170 μm. This thickness is in the order of commercially available PTFE membrane thickness (100–250 μm) used in other kind of devices, and much smaller than other porous proposed macrocapsules with 600 μm wall thickness^21^.

The devices were designed with a disc shape of 22 mm external diameter, 20 mm internal diameter and interior cavity of 1.5 mm height, as shown in Fig. 1B. The interior cavity height would allow enclosing no more of three close-packed layers of microencapsulated cells (∼400 μm size), assuring that all the microcapsules are close to the device membranes, where the oxygen diffusion would be larger. A central pillar was included to avoid the membranes deformation by external pressure, which could break the microcapsules.

Synthetic polymers are especially interesting for implant development, compared to natural polymers, given the lower stimulation of the immune system response, which would help avoiding the implant rejection by the host’s immune system and the low batch to batch variability^29–31^. However, synthetic polymers might show cytotoxicity problems, creating an important foreign body response when using them in implantable devices in direct contact with cellular environments. The devices were fabricated in polyamide because it has demonstrated high biocompatibility, flexibility and excellent mechanical properties. Polyamides are macromolecules with repeating units linked by amide bonds. Polyamide is commonly used in scaffolds and implants^31^ especially for bone reconstruction^32,33^, and is already approved by the U.S. Food and Drug Administration (FDA). Beside the long-lasting tensile strength and high elasticity, polyamide is compatible with the standard sterilization processes (autoclave), cause minimal tissue reactivity and is able to prevent bacterial transmission^31^. It presents as well a very good chemical resistance, which ensures that possible drugs do not promote the material’s degradation. Using polyamide allow the fabrication of flexible and durable thin membranes, which tolerate increasing the membrane porosity without compromising the macrocapsule mechanical stability. Polyamide can be easily manufactured by different technologies, including 3D printing.

Among various rapid prototyping technologies and 3D printers, selective laser sintering (SLS) has been found to be advantageous for devices fabrication due to its ability to process a wide range of biocompatible and biodegradable materials^34^, including polyamide. Besides being a rapid prototyping technique, SLS tolerates a large production in comparison with other rapid prototyping techniques with worse scalability. Usually, low cost rapid prototyping techniques are limited by the maximum resolution at both perpendicular and lateral dimensions. To circumvent this issue, porosity patterns with dimensions below or in the order of the laser spot size and the instrumentation lateral resolution were chosen, looking to achieve final pores with irregular shapes and dimensions below the system resolution. Before the device fabrication, different porosities patters were designed to test the optimum pore distance and size to avoid the complete pore blocking during the fabrication process, or the fabrication of large pores that would lead to cells microcapsules leaking. Figure 1C shows the test pattern performed, where it can be clearly seen the different final results depending on the selected pore size and pitch.

**Figure 2:**
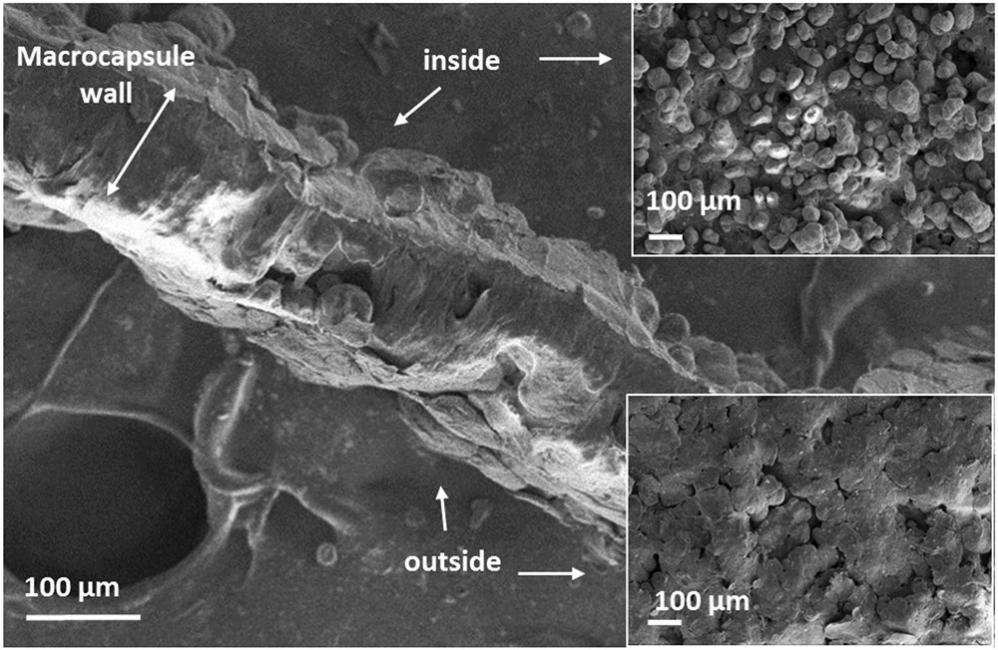
SEM image of a cross-sectioned macro-device wall (membrane), and the corresponding SEM images (top view) of the inner and outer side of the membrane.

Three different groups of devices, with different pore sizes and pitch, were fabricated and tested (as shown in Fig. 1C): D1, based on the fabrication of continuous sintered layer with no pattern, with a porosity related to the defects produced during the sintering process of a single layer; D2, based on a design pattern of square pores of 300 × 300 μm, with a pitch of 300 μm; and D3, based in a pattern of square pores of 300 × 300 μm, with a pitch of 400 μm. The final porosity achieved in each device was checked by optical microscopy (Fig. 1C) and scanning electron microscopy (SEM) (Fig. 2). The devices were cut in half to analyze the porosity of a single membrane. The optical images in Fig. 1C show an evident increase in the pore size and pore density from D1 to D3. Increasing the pitch produces porous patterns closer to the initial design (still with a mean pore size smaller than the given value).

The pore size histograms were obtained by optical image analysis (Image J software). The histograms show a pore mean length of 31 ± 15 µm for D1 devices (maximum length of 74 µm), 40 ± 26 µm for D2 (maximum length of 101 µm) and 81 ± 5 µm for D3 (maximum length of 191 µm). The morphological study performed by SEM shows the induced-fusion of the polymeric particles, and the imperfections (pores) left after the sintering process of a single layer. Figure 2 shows a cross-section of the membrane. A clear difference is observed between the inner and the outer side of the membrane. In the outer side, the polyamide microparticles are completely fused forming a continuous porous layer along the cross-section, while in the inner side, the microparticles are only partially fused, keeping their round shape.

**Figure 3:**
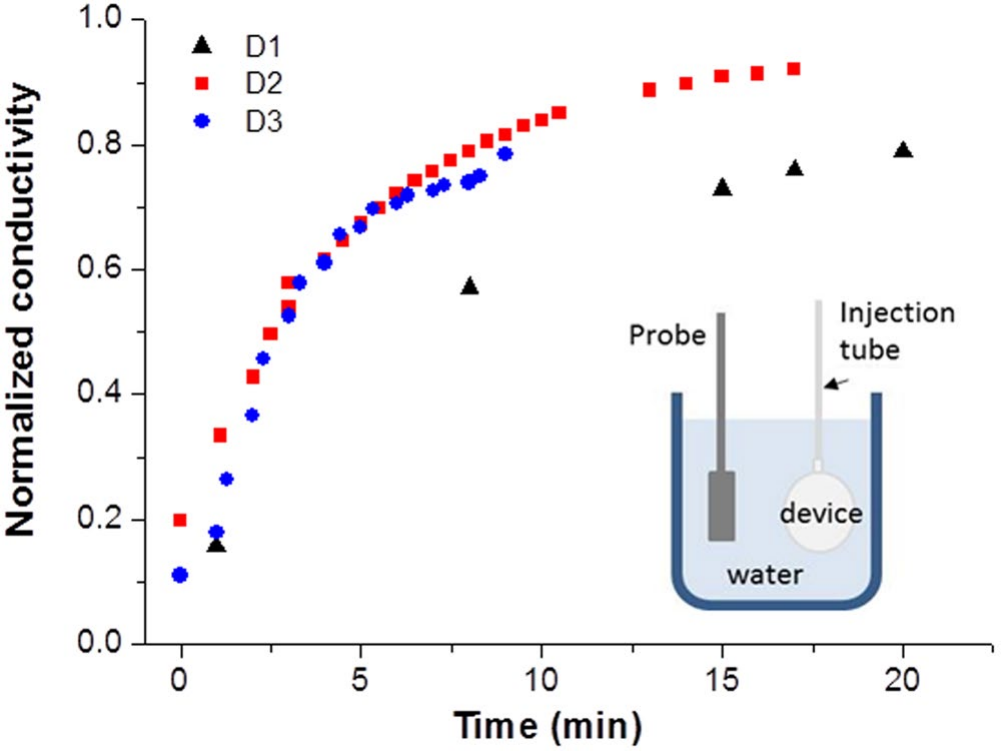
Normalized conductivity obtained during the diffusion measurements for the three groups of devices.

The diffusion of nutrients thought the macrocapsule membranes would be driven by the concentration gradient. Diffusion measurements were performed to determine the solute exchange through the macrocapsule device. We measured the diffusion of concentrated PBS solution, loaded into the macrocapsules, by measuring the conductivity change produced on an external bath. During the measurements, the syringe used to fill the devices was maintained connected. The conductivity results in Fig. 3 show no difference on the ionic diffusion between D2 and D3 porous devices. In contrast, slower diffusion occurs in the case of device D1. These results suggest that device D1 would have the worst performance in terms of nutrients diffusion and, therefore, in cell viability.

### Biological evaluation of medical devices

**Figure 4:**
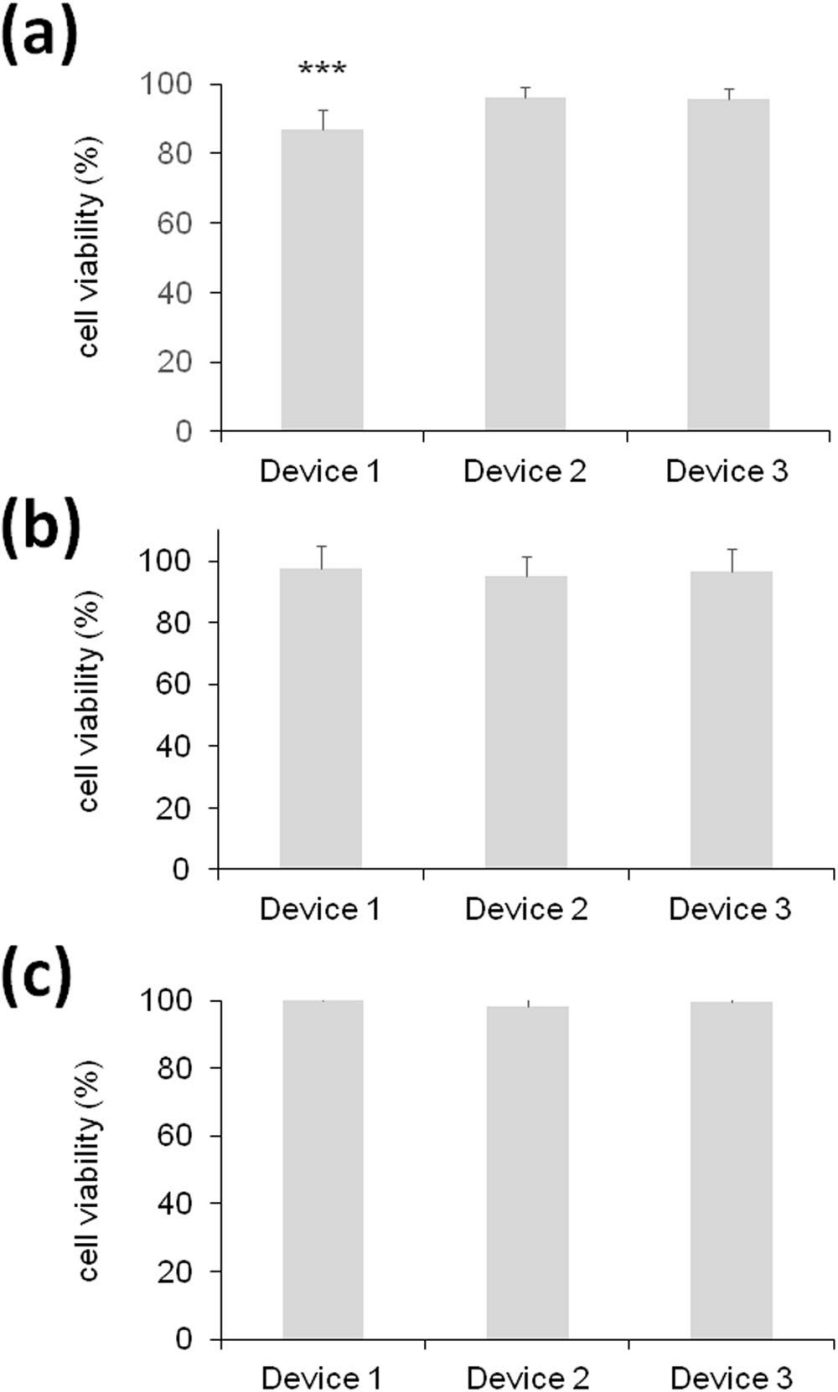
Biological evaluation of the three medical devices according to the ISO standards on mouse L929 fibroblasts assessed by the MTT toxicology assay. (**a**) Direct contact assay. (**b**) Indirect assay using conditioned media. (**c**) Adhesion assay. ***p < 0.001 compared with device 2 and 3.

In general, for tissue engineering applications, a successful scaffold should possess an adequate porous microstructure and the correct mechanical integrity together with good biocompatibility^35^. The ISO 10993 series of standards is recognized in both the European Union and United States. According to the described international standard ISO 10993-5, we performed three different kinds of assays. On the one hand, we studied the direct contact cytotoxicity of the devices on the L929 cell line. As it is shown in Fig. 4a, the percentage of live cells after contacting these devices (direct contact test) was very high in all cases (above 80%), although statistically significant differences were observed among the three different devices. When the macrocapsule had a continuous membrane (D1), the number of dead cells was around 15% while the other two devices with a higher pore showed lower values, in the order of 5%. On the other hand, we also studied the cytotoxicity provoked by the contact of the leachate (medium that had been in contact with each of the devices, indirect test) on the cells (Fig. 4b). In this case, no significant differences were observed among the devices and we could measure a near to 100% viability. In accordance with the ISO assessment of the results, a reduction on cell viability by more than 30% is considered as a cytotoxic effect or a cytotoxic potential. Therefore, we can conclude that our tested macrocapsules, independently of the pores of their membrane, do not present a remarkable cytotoxicity. Finally, we also performed an adhesion evaluation based on the MTT assay with the same kind of cells. As represented in Fig. 4c, 100% of the cells adhered to the material and no statistically significant differences were detected among the devices. This attachment of cells to the surface of the scaffolds represents the evidence of its biocompatibility^36^.

### Cell microencapsulation within alginate 3D matrices and macrocapsule entrapment

When cells encapsulated in 3D microcapsules are implanted in a patient, there is a little chance for individual cells to escape from the system as far as it has been covered with a semipermeable membrane as in alginate-PLL-alginate (APA) microcapsules^37^. However, there are some types of cells that, because of the way they grow inside the microcapsules, tend to form cell clusters that might compromise the viability of the cells^38,39^.

This fact might compromise the therapeutic purpose of the implant or, even more significantly, it could jeopardize the biosecurity of the therapy as, in the long term, the microcapsules could explode. Therefore, we developed a double biosecurity system that, in the event of cells forming agglomerates that are able to get out of the microcapsules, will reduce the possibility of cell clusters escaping from the macro-device. The first barrier would correspond to the microcapsule itself that has been covered by a double membrane (PLL and diluted alginate). The second barrier would be offered by the macrocapsule device in where the microcapsules are inserted. Also, these macrocapsule devices would allow the extraction and renewal of the encapsulated cells without the need of going through a major surgery. Undoubtedly, the viability of cells inside the APA microcapsules should be preserved to promote the implant functionality. Also, such double encapsulation system must enable both intimate communication between the entrapped cells, and the movement of exterior nutritional products that support the stable survival of the cells entrapped in the microcapsules.

We produced APA microcapsules containing either C_2_C_12_-EPO myoblasts or BHK-VEGF fibroblasts. These cell lines have successfully been used in preclinical models using different encapsulation systems for the secretion of recombinant proteins^40–43^. Also, in our experience, these two types of cells grow without problems inside alginate scaffolds and can be used either for *in vitro* or *in vivo* experiments without losing their functionality^44^. Once the cells were encapsulated in APA microcapsules, they were inserted into the three different macrocapsules and maintained in culture media in order to test the effect of membrane porosity on the outcome of C_2_C_12_-EPO and BHK-VEGF cells. As mentioned, the physical characteristics of the porous membrane might be a critical parameter for the survival of encapsulated cells. In fact, the rate of passive molecule diffusion across the membrane (inward diffusion of oxygen and nutrients and outward release of therapeutic factors) will be related to its porosity. At different time points over a month, devices were opened and the microcapsules were retrieved in order to perform an accurate assessment of the viability of encapsulated cells as this is essential for predicting the potential long-term secretion profiles of their therapeutic products.

**Figure 5:**
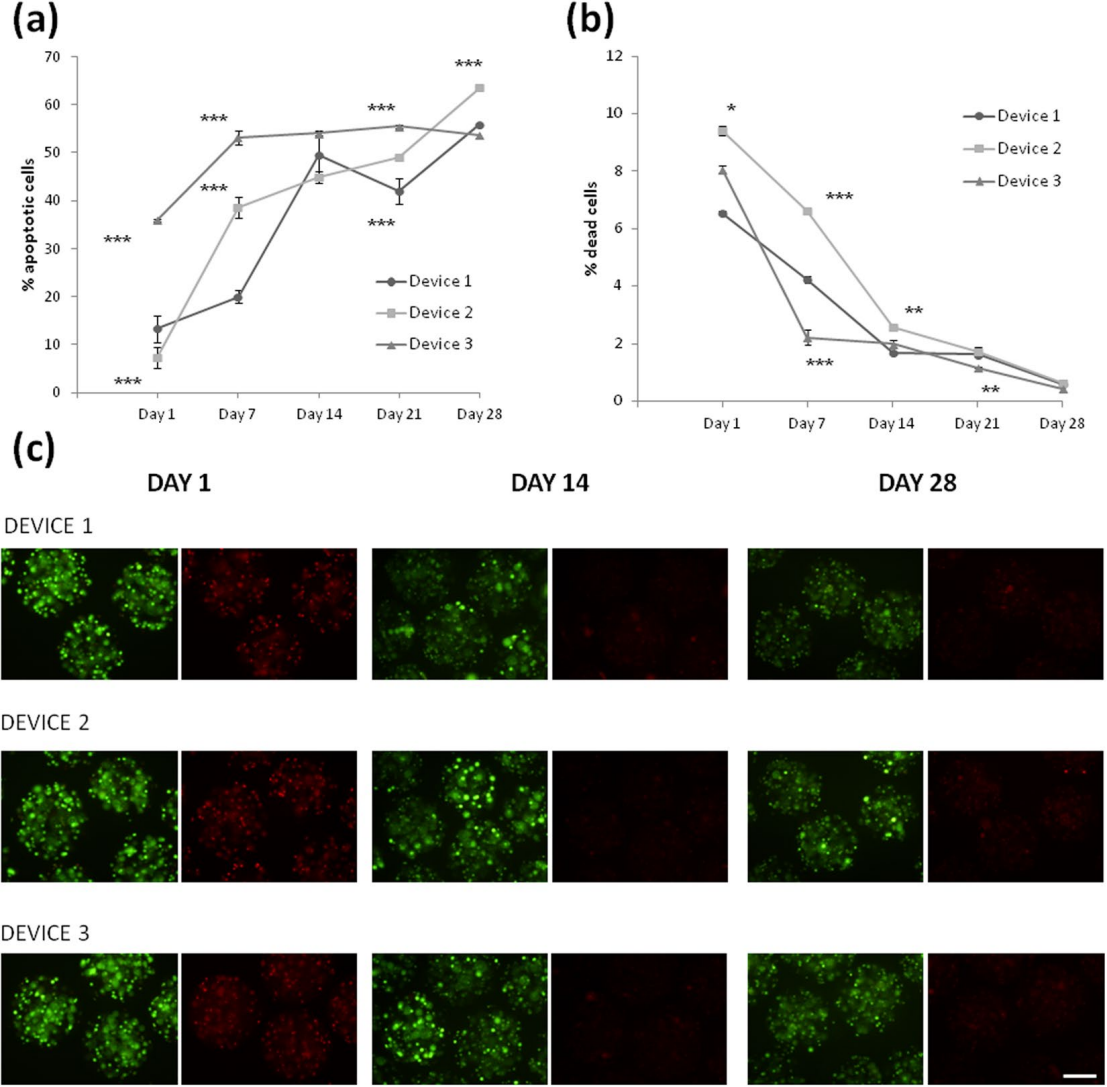
Effect of macro-devices’ porosity on the viability of encapsulated C_2_C_12_-EPO myoblasts within APA microcapsules. (**a**) Early apoptosis analysis by annexin/PI staining and (**b**) live/dead analysis by calcein/ethidium staining assessed by flow cytometry. (**c**) Fluorescence microscopy images of calcein/ethidium staining from microcapsules containing C_2_C_12_-EPO myoblasts embedded in the three studied devices. Scale bar 200 μm. *p < 0.05, ** < 0.01 and ***p < 0.001 compared with device 1 at the same time point.

First, we quantified by flow cytometry the percentage of apoptotic cells in the three devices every week. As seen in Fig. 5a, the percentage of apoptotic C_2_C_12_-EPO myoblasts was very low in D1 and D2 and surprisingly 3 times higher in D3. After the first week, this percentage increased dramatically in D1 and D2. However, cells micro and macroencapsulated in D3 showed a stable number of apoptotic cells very similar all over the whole monitorization process. In any case, the final amount of apoptotic myoblasts was around 55% in the three macrocapsules. This result is in accordance with previous published studies in which similar levels of apoptotic cells were detected in encapsulated C_2_C_12_-EPO myoblasts without using the external macro-devices 3 weeks after encapsulation. Therefore, it seems that, except for D3, which showed higher levels of apoptotic cells at day 1, the use of this double-encapsulation system does not affect the results on this flow cytometry assay^45^.

In parallel, we also quantified the percentage of dead cells at the same time points (Fig. 5b). This percentage was also low after encapsulation; it was reduced noticeably by the end of the first (D3) or second (D1 and D2) week and keep on falling, on the three devices, until the end of the study, where the final percentage of dead cells was less than 1%.

Accordingly, although this is a qualitative assay, the DNA integrity was also microscopically analyzed. Fluorescence microscopy images of calcein/ethidium staining confirmed the results quantified by flow cytometry showing a higher number of live cells (intracellular endogenous esterases hydrolyzed calcein-AM and turn it to fluorescent green) than dead cells (apoptotic and necrotic cells, where the ethidium bromide stained the fragmented DNA, in fluorescent red) and a reduction on the number of dead cells from day 1 to the end of the study (Fig. 5c). The higher apoptosis of cells during the first days might be as well related to the stress produced on the cells during the macroencapsulation process, due to the pressure applied to inject the microcapsules inside the devices.

**Figure 6:**
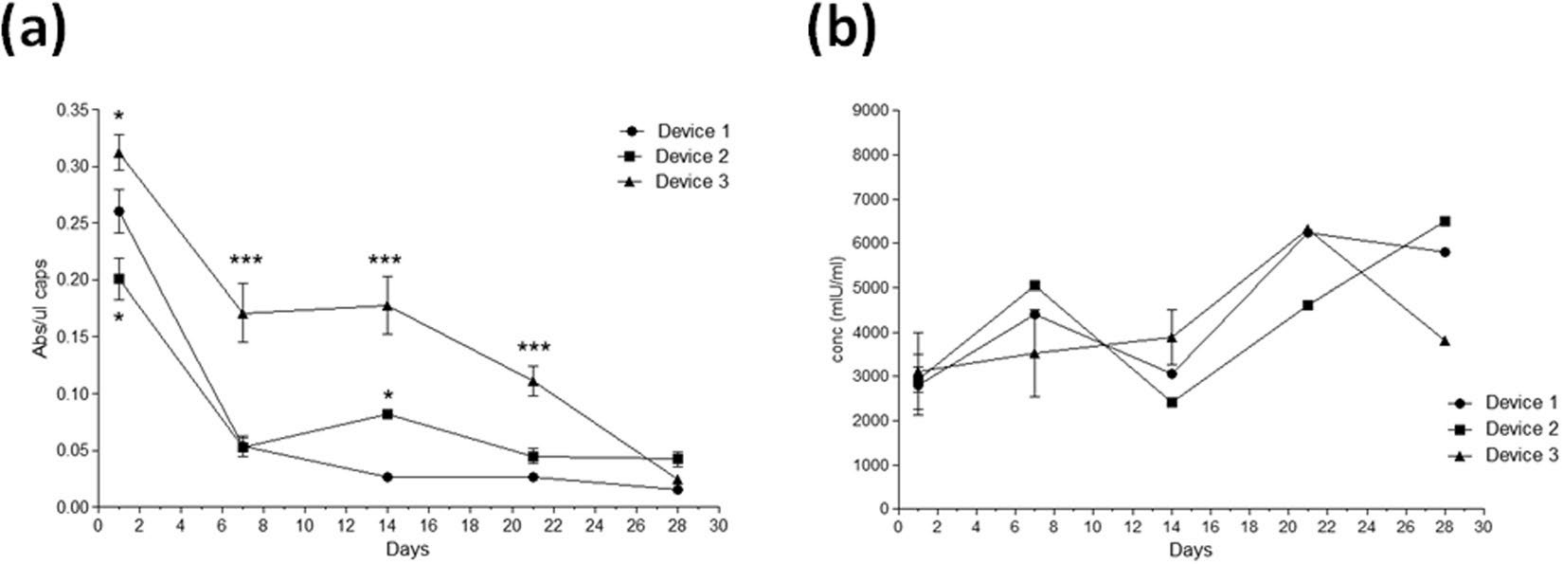
Effect of macro-devices’ porosity on the viability of encapsulated C_2_C_12_-EPO myoblasts within APA microcapsules. (**a**) Metabolic activity measured by the CCK8 assay. (**b**) Erythropoietin (EPO) secretion into the media. *p < 0.05 and ***p < 0.001 compared with device 1 at the same time point.

In order to analyze if encapsulated cells remained metabolically active inside the devices, the CCK8 assay was carried out. Results showed that the metabolic activity of encapsulated C_2_C_12_-EPO cells was reduced importantly on D1 and D2 (Fig. 6a). However, in D3, after a first 45% reduction step on the first week, the metabolic activity of the cells remained higher than in the other two devices. Nevertheless, by the end of the study, the activity of cells on the three macrocapsules was very similar and much lower than the day after encapsulation. Comparing these metabolic activity levels with the same cells encapsulated only in APA microcapsules, it can be appreciated that they are between 2 and 3 times higher, depending on thee macro-device, 24 hours after encapsulation^45^. Probably, the longer and more complicated process that must be performed for assembling this double-encapsulation structure accounts for higher levels of stress and, therefore, also higher levels of metabolic activity in the present study, mainly during the first week after encapsulation.

However, once the stress from the double-encapsulation procedure disappears, the metabolic activity levels are very low in all the devices. It is important to mention that this assay can also reflect the viable cell number in proliferation. Therefore, it would mean that the rate of proliferation inside this double system is even lower than inside APA microcapsules. Previously, it has been proved that the different content on guluronic and mannuronic acid of the alginate hydrogels has an important effect on cell proliferation and metabolism. For instance, experiments with murine insulinoma bTC3 cells found that alginates with high guluronic content, as the one that we have used in this project, inhibited cell growth, leading to decreased metabolic activity^46–48^. In addition to encapsulating the cells in alginate microcapsules, we have introduced them into supplementary macro-devices that have shown to reduce even more the metabolic activity of the cells. This could mean that cells are not comfortable enough inside our double security system because of a problem of oxygen and/or nutrients diffusion to the inner core of the microcapsules. This might be due to an excess of entrapped microcapsules inside the device, in a highly packed configuration, complicating the diffusion of oxygen and nutrients through the voids let between them. It could also reflect that cells need a period of time to acclimatize to the new environment and when achieved, they remain in a quiescent state with a low metabolic demand and mitotic activity^49,50^. It is likely that the hypoxia inside the devices affect myoblasts proliferation. In fact, hypoxia-inducible factors, such as hypoxia-inducible factor 1-alpha (HIF-1α), inhibit cell-cycle progression and act as master regulators of genes that allow adaptation to hypoxic conditions. Target genes include VEGF, erythropoietin, glucose transporters, and other factors critical to vascularization, metabolic regulation, cell multiplication and survival^51,52^. Undoubtedly, the metabolic activity reduction could compromise the therapeutic use of these devices in the clinic. However, the most critical aspect on the encapsulation therapy is the production of the required therapeutic protein and, more significantly, if these proteins are released both from the micro and macrocapsule to the culture media because the hypoxia-induced cell-cycle arrest is temporary and cell proliferation will restart when normoxia is restored^53^. In fact, a reduction on the proliferation rate, as far as the synthesis of the therapeutic product is enough for the therapy, is an advantage as there will be a lower risk of exploding the microcapsule and, in some cases where cells can grow forming cell-clusters, escaping from the whole macro-device.

As shown in Fig. 6b, the amount of EPO released from the three devices was very similar the day after encapsulation (around 3000 mIU/ml). At the end of the study, the concentration of protein detected on the culture media was higher on D1 and D2 than on D3. Importantly, it seems that the notorious reduction on cell metabolic activity has not a parallel impact on the amount of protein that is synthesized by these genetically modified myoblasts. Previous studies of alginate encapsulated C_2_C_12_-EPO myoblasts, without the external device, have shown lower production levels under the same sampling conditions. Therefore, the double-encapsulating system does not seem to imply a problem for the diffusion of therapeutic products. In fact, even from day 1 after encapsulation, levels of EPO in this study are 3 times higher than the previously published results^45^. It could be plausible that the paracrine stimulus that cells receive inside this system is higher as a consequence of capsules being more compactly packed. Similarly, other authors have reported a relative constant release of VEGF from NIH 3T3 fibroblasts encapsulated in 2% low viscosity alginate over a period of study of 21 days^54^. Likewise, neuronal stem cells encapsulated in alginate with high guluronic content have enhanced their secretion of neurotrophic factors^55^. This demonstrates the high capacity of the C_2_C_12_ myoblasts to adapt to difficult conditions of oxygen restriction inside macro-encapsulation devices, while they are able to maintain the production of high levels of therapeutic protein. Also, human mesenchymal stem cells (hMSCs) have shown to respond positively to hypoxia and nutrient deprivation^56,57^.

The same study was done for the VEGF producing BHK cells, to test both the suitability of the macrocapsule devices and the capability of the different cells lines to adapt to the experimental conditions given inside the macrocapsules. The VEGF-producing cells are especially interesting because VEGF has demonstrated to promote the scaffolds and implants vascularization^19^. They could be used in combination with some other type of cells or, in a first step of the therapy (pre-vascularization of the system prior to microencapsulated therapeutic cell insertion^58^, in order to induce an enhancement of the vascularization and perfusion around the devices. This strategy would help overcoming the low oxygen availability and reaching the therapeutic protein to the circulatory torrent in an easier and faster way^41,59^.

**Figure 7:**
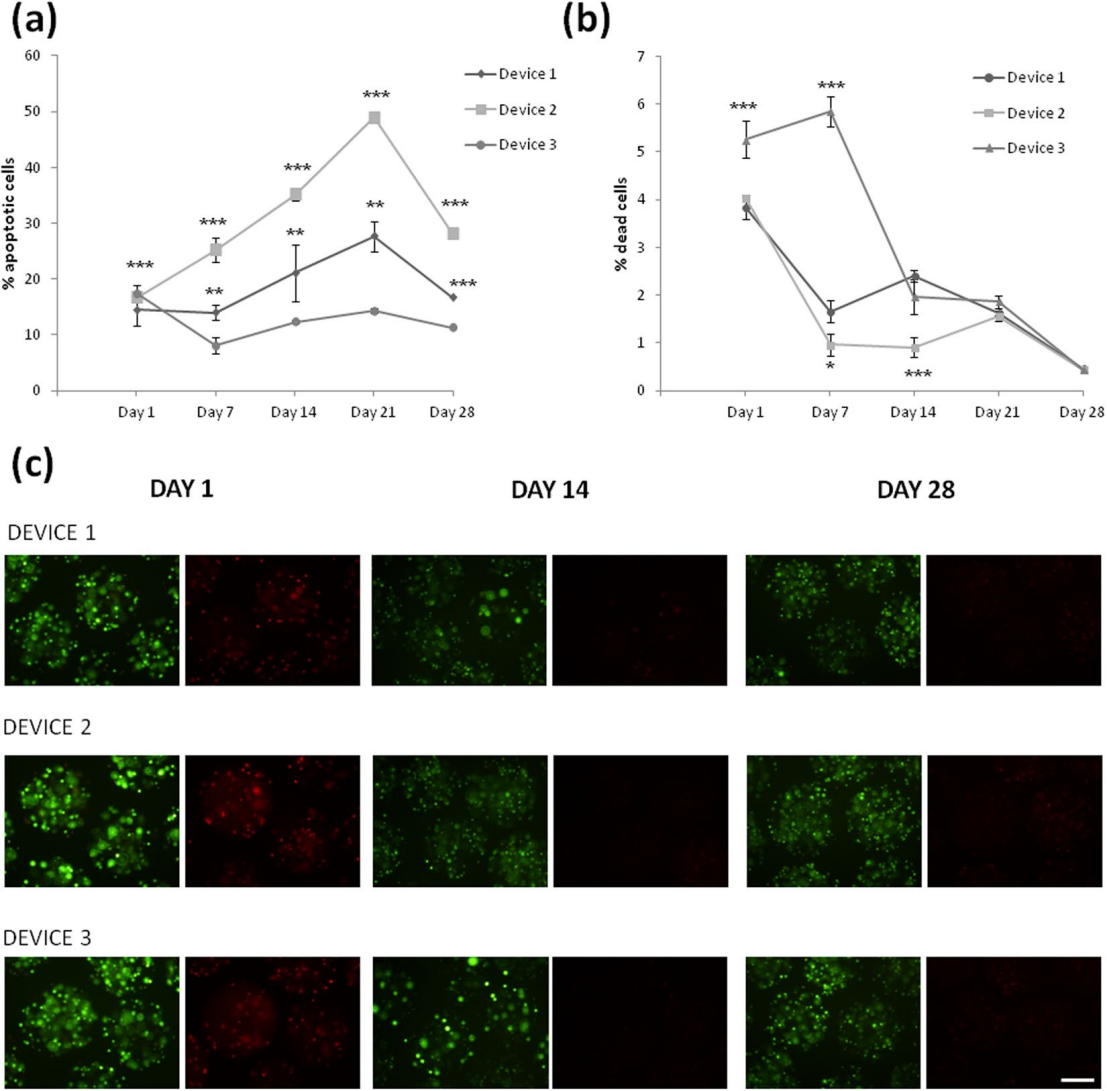
Effect of macro-devices’ porosity on the viability of encapsulated BHK-VEGF fibroblasts within APA microcapsules. (**a**) Early apoptosis analysis by annexin/PI staining and (**b**) live/dead analysis by calcein/ethidium staining assessed by flow cytometry. (**c**) Fluorescence microscopy images of calcein/ethidium staining from microcapsules containing BHK-VEGF fibroblasts embedded in the three studied devices. Scale bar 200 μm. *p < 0.05, ** < 0.01 and ***p < 0.001 compared with device 1 at the same time point.

For the BHK-VEGF cells, the cytometric analysis showed a number of apoptotic cells very similar in all the devices (around 15%) the day after encapsulation. This number of apoptotic cells increased along the days, especially in device D2, being the macrocapsule D3 which showed the lowest percentage of apoptotic cells with similar apoptosis percentage all over the study (Fig. 7a). Comparing these results with the ones obtained with the C_2_C_12_-EPO myoblasts, the percentage of apoptotic cells is lower for the BHK-VEGF cells. Looking to both results, we can conclude that the device D3 showed more stable results over the 4 weeks of study. The number of dead BHK-VEGF cells is represented in Fig. 7b, showing low percentages of dead cells in every device (less than 6% at day 1). The profile of these results is similar to the one obtained with the myoblasts, showing a first significant reduction on cell dead after the first week and a more stable and lower percentages until the end of the study, with final levels below 1%.

Similarly, fluorescence microscopy images confirmed a progressive reduction on the number of apoptotic or necrotic red-stained cells along the time (Fig. 7c).

**Figure 8:**
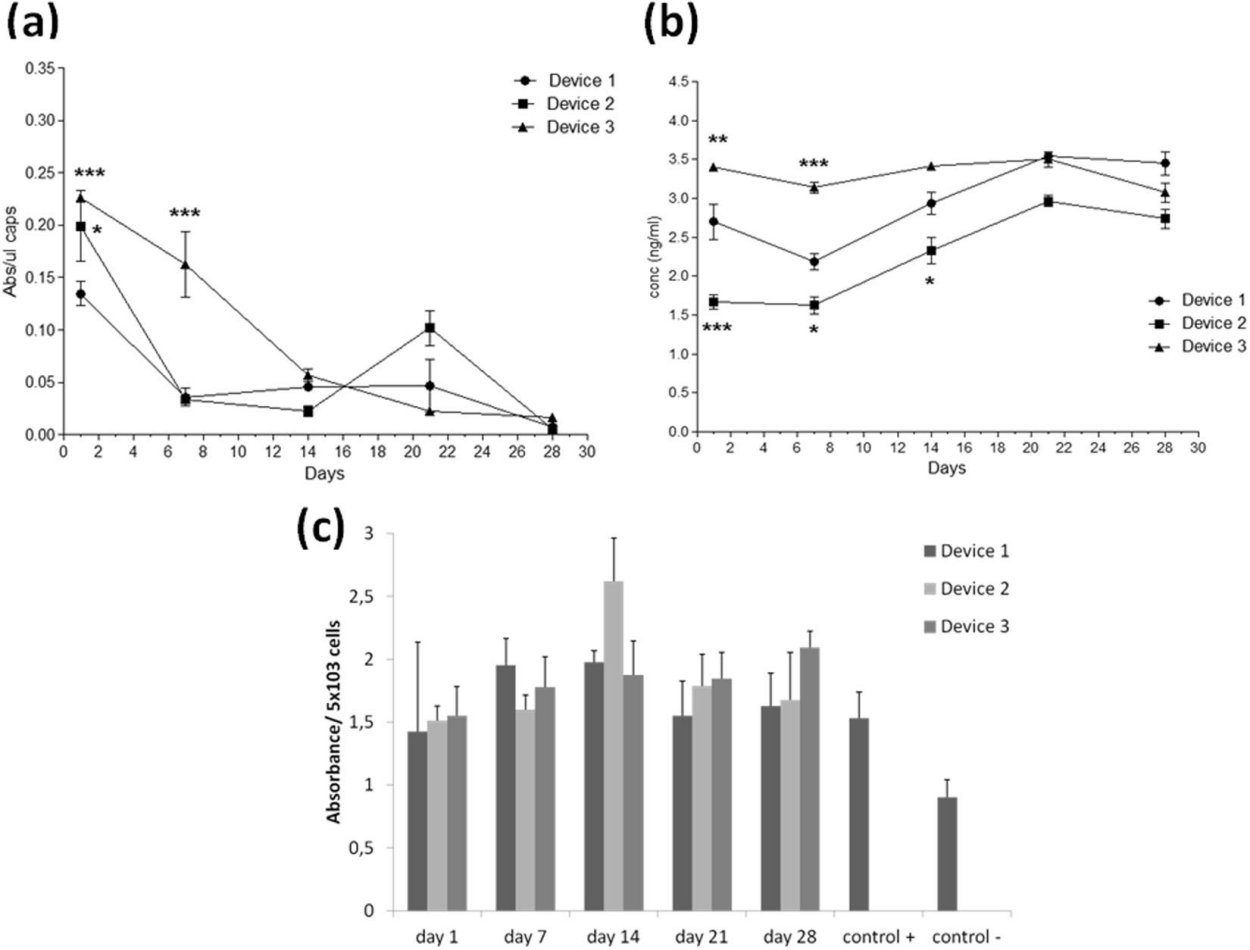
Effect of macro-devices’ porosity on the viability of encapsulated BHK-VEGF fibroblasts within APA microcapsules and VEGF’s bioactivity. (**a**) Metabolic activity measured by the CCK8 assay. (**b**) Vascular endothelial growth factor (VEGF) secretion into the media. (**c**) Assessment of VEGF bioactivity on HUVEC cells. *p < 0.05, ** < 0.01 and ***p < 0.001 compared with device 1 at the same time point.

Concerning the metabolic activity of the encapsulated BHK-VEGF fibroblasts, the activity is reduced drastically after the first week in D1 and D2, and later on, in D3, similarly to the results showed for C_2_C_12_-EPO myoblasts. The final activity in the three devices is significantly lower that at the beginning of the study, and equal in all the macrocapsules (Fig. 8a).

Fortunately, our results are not the anticipation to a bad therapeutic protein release profile. In fact, we observed that the VEGF protein was produced and released all over the study in a quite stable manner as shown in Fig. 8b. Interestingly, other authors have explained an increased VEGF secretion by the relative hypoxic environment inside the microcapsules^60^.

Once the release of VEGF from the encapsulated cells was confirmed, it was also important to determine whether the released VEGF remained bioactive, which was accomplished by testing its effect on HUVEC cells (Fig. 8c). All the samples of this protein released from any of the devices proved to be active all over the four weeks of the study at similar levels and above the positive control, which consisted on complete media supplemented with recombinant VEGF.

## Conclusions

We have combined the microencapsulation technology with the use of an external macro-device fabricated by SLS, as an approach to maintain the microcapsules at the implantation site, and improve the biosecurity of this technology. The use of macro-devices made with synthetic polymers in combination with naturally derived polymers used in the cell microencapsulation technology might have several advantages when translated into the clinical practice, as the former polymers have more dimensional stability, mechanical integrity and can be produced with well-defined microporosity. Large pores (>50 µm) polyamide macrocapsules with three different porosities, and different diffusion flow rates, were fabricated and tested. The macro-devices showed a very low cytotoxicity profile which makes them useful for clinical applications.

When combined with microencapsulated cells (manufactured alginate-poly-L-lysine microcapsules containing either erythropoietin producing C2C12 myoblasts or VEGF producing BHK fibroblasts), the macro-devices have shown to allow the diffusion of enough oxygen and nutrients in order to keep cells synthesizing therapeutic proteins, in spite of a metabolic activity reduction. Thus, the three tested macro-devices have proved to be suitable microencapsulated cells carriers, which provided a good cell viability, DNA integrity and therapeutic product production. Among them, device D3 showed the best performance in terms of cell viability, but no differences were observed on the metabolic or therapeutic protein release profile.

Therefore, the blending of these two macro and micro scaffolds that we have generated represents an innovative cell encapsulation approach that can provide a structural support to microcapsules so they can be maintained in an exact location in the body, and, at the same time, act as a double security system that allows the replacement or retrieval of the implanted encapsulated cells in an ease and minimum invasive way without removing the device. Thus, this system could be an advance towards the clinical application of the cell encapsulation technology thanks to its high immunoisolation capability and biosecurity characteristics.

## Methods

### Macrocapsule fabrication

The devices were initially designed with a computer aided-drafting (CAD) program (Autodesk Inventor), and fabricated by SLS (Fig. 1a,b). In the SLS process, 3D computer images are sectioned into thin 2D layers and the 3D objects are built layer-by-layer to the required size, shape and internal structure by laser-induced fusion of small particles of ceramic, metal or thermoplastic powders. The used laser sintering instrumentation (Formiga P 100 from EOS GmbH) was equipped with a CO2 laser (10.6 μm wavelength, <0.5 mm spot size, and 440 mm focus distance) with a maximum power of 30 W and maximum scanning speed up to 5 m/s. The minimum layer thickness is 100 µm on z and about 600 μm on x and y. In this case, polyamide PA2200 (EOS GmbH) was used for the devices fabrication.

PA 2200 is in compliance with the EU Plastics Directive 2002/72/EC for the use with food at contact conditions up to 24 hours at 20 °C, and parts made of PA 2200 are biocompatible according to ISO 10993-1. The processing parameters applied for the polyamide sintering were 25 W of laser power and 5 m/s of scan speed. The devices were sterilized by autoclave before their used.

### Electrolytes diffusion through macrocapsule membranes

The diffusion of electrolytes through the macrocapsule device was tested by measuring the conductivity change (CRISON GLP31) of a water bath (25 ml) where the devices, filled with 500 µl of phosphate-buffered saline (PBS) 100 mM, were immersed. The hydrophobic macrocapsules were able to retain the PBS solution during a short period, before being introduced into the water bath. Water and PBS conductivity were 0.8 µS/cm and 15.39 mS/cm, respectively. A final conductivity in the water bath of 380 µS/cm is expected (25 ml of water with 500 µl of PBS) after reaching the steady-state. This value was used to normalize the measured conductivity for each device.

### Cell culture conditions for cell encapsulation

Murine C_2_C_12_ myoblasts genetically engineered to secrete EPO (C_2_C_12_-EPO) and Baby hamster kidney (BHK) fibroblasts modified to secrete VEGF (BHK-VEGF) were grown in T-flasks with Dulbecco’s modified Eagles’s medium (Gibco) supplemented with 10% FBS, 2 mM L-glutamine (Gibco) and 1% antibiotic/antimycotic solution (Gibco) at 37 °C in humidified 5% CO_2_/95% air atmosphere. Cells were passaged every 2–3 days.

### Biological evaluation of medical devices

Three different assays were conducted to evaluate the cellular toxicity of the studied devices following the international standards covered in ISO 10993-5: direct and indirect cytotoxicity, and adhesion assays. Mouse L929 fibroblasts were used to perform all experiments. Cells were cultured in EMEM media (ATCC) supplemented with 10% Fetal Calf Serum (Sigma), 1% penicillin/streptomycin (Invitrogen) and 4 mM glutamine (Sigma) at 37 °C in humidified 5% CO_2_ atmosphere. In all the experiments, cells were seeded at a density of 3.123·10^4^ cells/cm^2^. In the direct and indirect cytotoxicity assays cells were seeded and maintained in culture for 24 h (less than 1 doubling period) in order to form a semi-confluent monolayer. Then, they were exposed directly to the devices placing them onto the cell monolayer, or indirectly by adding conditioned media (media that has been in contact with the device for 24 h) on the cell monolayer. On the other hand, in the adhesion assay cells were seeded at the same cell density onto the devices. After 4 and 24 h, cell viability was measured in the adhesion assay and in the direct and indirect assays respectively using the 3-(4,5-dimethylthiazol-2-yl)−2,5-diphenyltetrazoliumbromid (MTT) *in vitro* toxicology assay kit (Sigma) following manufacturer’s recommendations. In the adhesion assay, cells seeded directly onto the culture plate were used as the absolute value of optical density in the untreated blank. Instead, in the direct and indirect tests, cells with no device exposure were used as controls. The absorbance was recorded using the Infinite M200 microplate reader (TECAN Trading AG) at 570 nm with reference wavelength set at 650 nm. Cell viability was calculated using the following equation: Cell viability = (testing sample OD_570_/untreated blank OD_570_) × 100. Four independent experiments were conducted with three replicates each.

### Cell encapsulation in 3D alginate microcapsules

Sterile 1.5% (w/v) ultra-pure low-viscosity and high guluronic (LVG) sodium alginate solution (FMC Biopolymer, Norway) was prepared by dissolving it in a 1% (w/v) mannitol solution and filtered through a 0.20 µm syringe filter (Millipore, MA, USA). Either C_2_C_12_-EPO or BHK-VEGF cells at a density of 5·10^6^ cells/ml were harvested and resuspended into the aforementioned sterile 1.5% alginate solution. Microcapsules were prepared using an electrostatic droplet generator (Nisco Engineering AG, Zurich, Switzerland). Briefly, the cell suspension was extruded through a sterile 0.17 mm inner diameter needle using a 10 ml sterile syringe with a peristaltic pump into a calcium chloride solution (55 mM). The resulting alginate beads were maintained in agitation for 10 minutes for complete ionic gelation. Then, microcapsules were successively chemically cross-linked with 0.05% (w/v) poly-L-lysine for 5 minutes (PLL; MW 15.000–30.000 Da; Sigma Aldrich, St. Louis, MO, USA) and then coated with 0.1% (w/v) LVG-alginate solution for 5 minutes. All alginate-poly-L-lysine-alginate (APA) microcapsules were prepared at room temperature, under aseptic conditions. The diameters (380 ± 20 µm) and overall morphology of microcapsules were characterized using an inverted optical microscopy (Nikon TSM).

### Loading of cell-containing APA microcapsules in the macrocapsules

After the microencapsulation process, 500 µl of microcapsules (2·10^6^ cells) suspended in 1 ml of culture media were loaded into the polyamide macrocapsule by slow injection through the macrocapsule inlet. The macrocapsule permeability allows the injection of the microcapsules in media, leaking the solution out of the device and retaining the microcapsules inside.

The devices containing APA microcapsules were maintained in culture media and, at different time points over a month, microcapsules were retrieved from the macrocapsules to assess the viability and metabolic activity. Also, protein secretion studies were performed on the conditioned media (supernatants) obtained from microcapsules-containing devices.

### Flow cytometry assays: early apoptosis and cell viability quantification

Early apoptosis of both types of cells (C_2_C_12_-EPO and BHK-VEGF) microencapsulated within APA microcapsules was quantified with the Annexin-V-FITC apoptosis Detection Kit (Sigma-Aldrich) once a week for a month post-encapsulation. 250 µl of microcapsules (10^6^ cells) retrieved from the macro-devices were incubated with alginate lyase (Sigma-Aldrich) for 30 minutes at 37 °C to release the embedded cells. Next, cells were rinsed twice with DPBS, resuspended in 10 mM HEPES/NaOH containing 0.14 M NaCl and 2.5 mM CaCl_2_ (binding buffer, pH 7.5) and stained with annexin V-FITC and propidium iodide for exactly 10 minutes at room temperature protected from light. Fluorescence was determined immediately with a BD FACS Calibur^TM^ flow cytometer (BD Biosciences). Unstained samples or samples stained only with annexin V-FITC or propidium iodide were used to establish the appropriate acquisition parameters for the analyzed samples.

For cell viability studies, the same samples and controls were quantified with the LIVE/DEAD^®^ Viability/Cytotoxicity Kit (Invitrogen™) at the same time points. After cells were released from the microcapsules, they were stained with 100 nM calcein AM and 8 µM ethidium homodimer-1 solution for 20 minutes at room temperature, protected from light. Fluorescence was determined immediately with a BD FACS Calibur flow cytometer^TM^. Unstained samples or samples stained only with 100 nM calcein AM or 8 µM ethidium homodimer-1 were ran as controls to establish the appropriate acquisition parameters for the analyzed samples.

### Cell viability qualitative determination by fluoresce microscopy

For cell viability determination under the microscope, samples were stained with the LIVE/DEAD^®^ Viability/Cytotoxicity Kit. First, microcapsules retrieved from the devices were washed four times in a test tube with DPBS and allowed to sediment. Then, they were stained with 0.5 µM calcein AM and 0.5 µM ethidium homodimer-1 in DPBS on 96-well plates at room temperature for 40 minutes in the dark. Next, samples were observed under a Nikon TMS microscope with the excitation/emission settings for calcein AM (495/515 nm) and ethidium homodimer (495/635 nm). At least three independent experiments were analyzed for each condition.

### Cell metabolic activity determination

The metabolic activity of encapsulated cells was determined weekly post-encapsulation. With this purpose, 5 µl of retrieved microcapsules per well were placed in 100 µl of medium on 96-well plates. Afterwards, 10 µl of Cell Counting Kit-8 (CCK-8) solution (Sigma Aldrich) were added to each well. Plates were incubated inside a humidified chamber for 4 hours at 37 °C and absorbance was read on an Infinite M200 (TECAN Trading AG, Switzerland) microplate reader at 450 nm with reference wavelength set at 650 nm. Wells containing culture media were used as negative controls. At least five wells were placed for each condition.

### EPO and VEGF secretion determination

For protein secretion determination, 6-well plates were plated with microcapsules-containing devices in 5 ml of media and supernatants were collected after 24 hours at different time points and frozen at −80 °C until they were analyzed. The amount of released EPO was assayed with the Quantikine IVD EPO Elisa kit (R&D Systems) and the VEGF release was quantified with the VEGF Elisa Development Kit (Peprotech). All samples and standards were measured at least in duplicate. Also, three independent experiments were analyzed for each experiment.

### Determination of VEFG activity

The biological activity of the VEGF released from the microcapsules was tested in the HUVEC (human umbilical vein endothelial cells) (PromoCell GmbH, Germany) cells because VEGF induces high proliferation in these cells^61^. HUVEC cells were maintained in endothelial cell growth medium (PromoCell GmbH, Germany) supplemented with a supplement kit and were cultured in special cell culture flasks (Corning CellBIND Surface) at 37 °C in humidified air with 5% CO_2_. After 2 or 3 passages, cells were seeded into 96-well poly-L-lysine-coated culture plates at a density of 5·10^3^ cells/well. After 24 h, the culture medium was removed and fresh media (without the supplement kit) was added and supplemented with one of these options: 0.8 ng/ml of VEGF released from BHK-VEGF containing microcapsules, 1 ng/ml of VEGF commercial solution as a positive control, or the released medium from empty microcapsules as a negative control. Cells were incubated for 72 h and cell proliferation was measured by the Cell Counting Kit-8 (CCK-8 assay) as explained previously.

### Statistical analysis

An analysis of variance (ANOVA) and post hoc analysis were performed to assess the statistical significance of the data collected. P < 0.05 was indicative of a statistically significant difference. All the data were analyzed with SPSS 13.0 software (Statistical Package for the Social Sciences, Chicago, IL, USA) or GraphPad Prism 5.01 (GraphPad Inc., San Diego, CA) and are presented as the means ± SD.

### Data availability

The datasets generated during and/or analysed during the current study are available from the corresponding author on reasonable request.
